# Comparing smoking habits and tobacco-related education between Canadian and Greek medical students

**DOI:** 10.1186/1617-9625-12-S1-A5

**Published:** 2014-06-06

**Authors:** Georgios-Marios Pantsidis, Dimitra-Iosifina Papageorgiou, Demosthenes Bouros

**Affiliations:** 1 Faculty of Medicine, Democritus University of Thrace, Alexandroupolis, 68100, Greece; 2 Faculty of Medicine, University of Thessaly, Larisa, 41110, Greece

## Background

According to a survey on Canadian medical students’ smoking habits and beliefs, the key results show that the prevalence of smoking among the future healthcare professionals is high and they lack of tobacco-related education [1]. Last year a similar survey was conducted at Democritus University of Thrace [2]. Its findings show that there is difference in smoking habits between the two students’ groups, but their tobacco-related education is equally poor.

## Materials and methods

In both researches participated undergraduate students who completed a questionnaire about their smoking habits, attitudes and education level towards tobacco cessation interventions.

## Results

The prevalence of cigarette smoking among Greek medical students is higher than the Canadians (24% vs. 3.3%). Although Canadian students smoke, also, other tobacco products (cigars, water pipe), the total prevalence is 15.3%. 65.5% of the Greek medical students report that they had ever tried cigarettes, but only 29.9% of the Canadian students make a same statement. Both students groups reported that they have moderate levels of education concerning tobacco-related subjects and cessation techniques. Only 8.1% of Greek and 10% of Canadian medical students report that they had ever received trainings in smoking cessation methods. Finally only a small percentage seems to be familiar with the cessation guidelines and only a few students are aware of the fact that they lack knowledge to help their patients cease smoking.

## Conclusions

The prevalence of cigarettes smoking among Greek medical students is significantly higher. Also the tobacco-related education in both countries is equally poor. It is desperately necessary to enhance the medical schools’ curricula with courses regarding smoking issues, since future physicians have a key-role in tobacco cessation and prevention.
